# CD40-targeted adenoviral GM-CSF gene transfer enhances and prolongs the maturation of human CML-derived dendritic cells upon cytokine deprivation

**DOI:** 10.1038/sj.bjc.6601225

**Published:** 2003-09-30

**Authors:** A G M Stam, S J A M Santegoets, T M Westers, C C Sombroek, J J W M Janssen, B W Tillman, A A van de Loosdrecht, H M Pinedo, D T Curiel, G J Ossenkoppele, R J Scheper, T D de Gruijl

**Affiliations:** 1 Department of Pathology, Free University Medical Center, PO Box 7057, 1007 MB Amsterdam, The Netherlands; 2 Department of Hematology, Free University Medical Center, PO Box 7057, 1007 MB Amsterdam, The Netherlands; 3 Division of Human Gene Therapy, Departments of Medicine, Pathology, and Surgery and the Gene Therapy Center, University of Alabama at Birmingham, AL 35294, USA; 4 Department of Medical Oncology, Free University Medical Center, PO Box 7057, 1007 MB Amsterdam, The Netherlands

**Keywords:** adenovirus, dendritic cell, CML, GM-CSF, CD40 ligation, immunotherapy

## Abstract

Vaccination with autologous leukaemia-derived dendritic cells (DC) presents an adjuvant treatment option for chronic myeloid leukaemia (CML). Here, we show that high-efficiency CD40-targeted adenoviral gene transfer of GM-CSF to CML-derived DC induces long-lived maturation in the absence of exogenous cytokines and may thus ensure protracted stimulation of CML-specific T cells upon vaccination.

It has become clear from clinical observations that chronic myeloid leukaemia (CML) is amenable to T-cell-mediated immune surveillance (Kolb *et al*, 1995). An immunotherapeutic approach may therefore provide a valuable adjuvant treatment option, especially in minimal residual disease settings.

Leukaemic dendritic cells (DC), capable of eliciting T-cell responses against CML targets (Choudhury *et al*, 1997; Eibl *et al*, 1997), are easily generated, carry all possible tumour antigens, and can be used for autologous vaccination (Ossenkoppele *et al*, 2003). Although it is still unclear how long DC should maintain their mature phenotype and survive *in vivo* in order to generate an effective antitumour T-cell response, it is generally accepted that a stable mature DC phenotype is required to prevent reversion to a possibly tolerogenic immature precursor state subsequent to adoptive transfer. In this light, introduction of the gene encoding granulocyte/macrophage-colony stimulating factor (GM-CSF) into CML-DC may be an attractive approach to both enhance and prolong antigen presentation and thus promote antileukaemic responses (Thomas *et al*, 1998). Recombinant adenoviruses (Ad) are attractive tools for gene transfer to DC, since they have been reported to enhance the immunostimulatory capacity of DC (Morelli *et al*, 2000). In addition, we previously reported that retargeting Ad to CD40 on monocyte-derived DC (MoDC) through a bispecific immunoconjugate could further increase DC maturation and simultaneously enhance gene transfer (Tillman *et al*, 1999). Here, we report on the utility of CD40-retargeted Ad to transduce CML-DC very efficiently, thereby reducing the viral dose needed for the desired level of transgene expression and simultaneously inducing phenotypic maturation as well as enhanced functional activity of the CML-DC. Moreover, we show that the introduction of GM-CSF into CML-DC by means of CD40-targeted Ad can significantly prolong the stability of the mature CML-DC phenotype.

## MATERIALS AND METHODS

After informed consent, heparinised blood samples were collected from patients with chronic-phase CML at diagnosis or during treatment with hydroxyurea and/or IFN-*α*. PBMC were isolated and CML-DC generated, phenotypically characterised, and tested for leukaemic origin by bcr-abl FISH, as previously described (Ossenkoppele *et al*, 2003).

Replication-deficient recombinant Ad-5 vectors and bispecific Ad-targeting antibody conjugates were constructed, produced, purified, and used for CML-DC infection as previously reported (Tillman *et al*, 1999). The expression of green fluorescent protein (GFP) and DC differentiation and maturation markers was assessed by FACS, T-cell stimulatory capacity by allogeneic MLR, and IL-12 p70 production by ELISA, all as described previously (Tillman *et al*, 1999). For the assessment of GM-CSF production by CML-DC (at 0.5 × 10^6^ ml^−1^), supernatants were tested 24 h postinfection using an ELISA according to the manufacturer's specifications (Biosource Int., Camarillo, USA).

Transduction efficiencies (%), mean fluorescence intensities (MF), and mean fluorescence Indices (MFI) were compared between test conditions using a paired, two-tailed Student's *t*-test. Differences were considered significant when *P*<0.05.

## RESULTS

**Figure 1:**
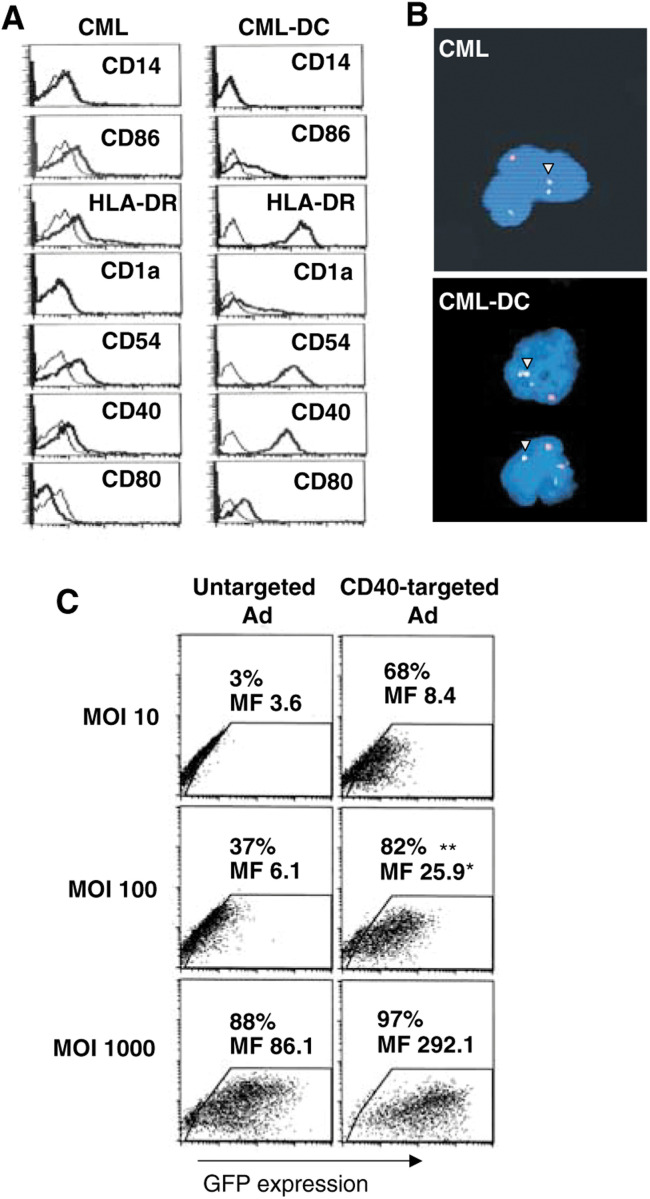
CML cells cultured with GM-CSF, IL-4, and TNF-*α* acquire DC phenotype and retain their leukaemic origin. Targeting Ad to CD40 on CML-DC significantly enhances gene transfer. (**A**) CML and CML-DC were stained with fluorescein- or phycoerythrin-conjugated antibodies and analysed by flow cytometry. CML-DC showed enhanced expression of CD1a, CD40, CD54, CD80, CD86, and HLA-DR. Thin lines represent the isotype control antibodies, bold lines represent staining with antibodies to the indicated antigen. Similar results were noted for nine other patients. (**B**) Cytospins were prepared from CML cells and CML-DC and an interphase t(9;22) FISH was performed. Most CML-DC showed both red (bcr) and green (abl) signals and one yellow (fused bcr-abl) signal (indicated by the yellow triangles). Nuclei were stained blue by DAPI. The results for one patient's uncultured CML cells and the corresponding CML-DC after culture (magnification × 400) are shown. (**C**) CML-DC were infected with Ad-GFP in the presence or absence of the Fab-anti-CD40 conjugate (a bispecific immunoconjugate consisting of a chemically linked Fab fragment of the S11 anti-Ad5 fibre knob mAb and the G28-5 anti-CD40 mAb; (Tillman *et al*. 1999). Cells were analysed by flow cytometry 48 h after infection; transduction efficiencies are given (in %) and the levels of GFP expression are depicted as MF intensities. One representative experiment out of four is shown. Significant differences were found for CD40-targeted *vs* untargeted Ad at MOI 100, based on results from four separate experiments; GFP transgene expression levels: ^*^P<0.05, transduction efficiency: ^**^P<0.01.

PBMC of chronic-phase CML patients were used to generate DC. After 14–21 days culture in the presence of GM-CSF (100 ng ml^−1^), TNF-*α* (2.5 ng ml^−1^), and IL-4 (1000 U ml^−1^), the cells displayed a typical DC morphology. This was accompanied by *de novo* expression of CD1a and CD80, and marked upregulation of CD86, CD54, CD40, and HLA-DR (Figure 1A

). To confirm the leukaemic origin of the generated DC, cells were analysed for the presence of the bcr-abl gene by FISH (Figure 1B).

FACS analysis showed the primary Ad docking receptor CAR to be absent, but the *α*v*β*3-integrin, facilitating Ad internalisation, to be present on the cell surface of the CML-DC (data not shown). To evaluate the susceptibility of CML-DC to Ad infection, CML-DC were transduced with Ad encoding GFP (Ad-GFP) at MOI ranging from 10 to 1000 plaque-forming units (PFU)/per cell. In line with their lack of CAR expression, CML-DC were relatively resistant to transduction with Ad, but transduction efficiencies and GFP transgene expression levels could be significantly increased by retargeting Ad to CD40 as an alternate docking receptor (Figure 1C).

**Figure 2:**
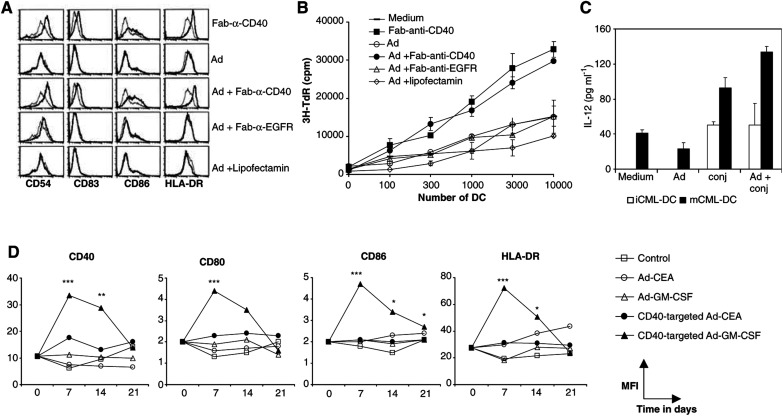
CD40-targeted Ad transduction of CML-DC results in maturation. Introduction of the GM-CSF gene by this means leads to a prolonged mature phenotype. CML-DC were incubated with the bispecific Fab-anti-CD40 conjugate or infected with Ad in the presence or absence of the Fab-anti-CD40 conjugate, a Fab-anti-EGFR control conjugate, or lipofectamin. (**A**) After 48 h, transduced cells were stained with fluorescein- or phycoerythrin-conjugated antibodies and analysed by flow cytometry. Thin lines represent the isotype control antibodies, bold lines represent staining with antibodies to the indicated antigen. (**B**) After infection, transduced CML-DC were used as stimulator cells in an allogeneic MLR with 0.1 × 10^6^ PBL per well as responder cells. ^3^H-thymidine incorporation was measured at day 5 as a measure for proliferation. (**C**) Uninfected CML-DC or CML-DC infected by untargeted or CD40-targeted Ad-GFP were cultured at 40 000 cells per 200 *μ*l for 24 h in the presence of 1000 U ml^−1^ IFN-*γ*, after which IL-12 (p70) production was assessed in the supernatants. Immature CML-DC (iCML-DC) or LPS-matured (for 1 h at 100 ng ml^−1^ LPS) CML-DC (mCML-DC) were thus tested. All results shown are representative of at least three independent experiments. (**D**) CML-DC were infected with Ad-GM-CSF in the presence of Fab-anti-CD40 for 1 h at MOI 100 and compared to mock-infected DC, DC infected with (un)targeted AdCEA, or DC infected with untargeted Ad-GM-CSF. The expression of the indicated activation markers was assessed at days 0, 7, 14, and 21 by staining with PE- or FITC-conjugated antibodies. Mean fluorescence indices for each marker are depicted. Results from one representative experiment (out of five) are shown. Indicated statistically significant differences are based on data from all five experiments: ^*^P<0.05, CD40-targeted Ad-GM-CSF *vs* Ad-GM-CSF alone; ^**^P<0.05, CD40-targeted Ad-GM-CSF *vs* CD40-targeted Ad-CEA; ^***^P<0.05, CD40-targeted Ad-GM-CSF *vs* both untargeted Ad-GM-CSF and CD40-targeted Ad-CEA. GM-CSF production levels were as follows (at 24 h postinfection, 5 × 10^5^ CML-DC^−1^ ml, *n*=5): control and Ad-CEA; undetectable; Ad-GM-CSF; 264 pg ml^−1^; range 18–635 pg ml^−1^; CD40-targeted Ad-CEA, 138 pg ml^−1^, range 0–274 pg ml^−1^; CD40-targeted Ad-GM-CSF; 7798 pg ml^−1^, range 2069–18446 pg ml^−1^.

To evaluate whether CD40-retargeted Ad infection was accompanied by maturation of CML-DC, as we previously showed for MoDC, we infected CML-DC with untargeted or CD40-targeted Ad, and compared both their phenotype and function with uninfected CML-DC, CML-DC infected by epidermal growth factor receptor (EGFR)-retargeted Ad (using a control immunoconjugate), or CML-DC infected by liposome-complexed Ad (lipofectamin-Ad), which facilitated high-efficiency Ad infection comparable to CD40 retargeted Ad. As shown in Figure 2A

, neo-expression of CD83 was induced and the expression of CD54, CD86, and HLA-DR was specifically enhanced upon CD40 targeting. These phenotypic changes were accompanied by a significantly enhanced T-cell-stimulatory capacity (Figure 2B) and the release of IL-12-p70 (Figure 2C).

To determine the combined effect of CD40 activation and GM-CSF production on the maturation and the stability of the DC phenotype over time, we transduced CML-DC with untargeted or CD40-targeted Ad-GM-CSF or Ad encoding the carcinoma-embryonic antigen as a control vector (Ad-CEA). The expression of a panel of maturation molecules was evaluated at several time points after infection. Optimal CML-DC activation was generally measurable 3 days after CD40-retargeted Ad-GM-CSF transduction and was only marginally higher as compared to the effects of CD40-retargeted Ad-CEA transduction (not shown). However, significantly elevated levels of CD40, CD80, CD86, and HLA-DR on CML-DC transduced by CD40-targeted Ad-GM-CSF could be maintained until days 7–14 as compared to CML-DC transduced by untargeted Ad-GM-CSF or CD40-retargeted Ad-CEA (Figure 2D). Indeed, without CD40-retargeted Ad-GM-CSF transduction, levels of DC activation markers dropped to basal levels within 7 days after cytokine deprivation (Figure 2D). Transduction of CML-DC with Ad-GM-CSF complexed to liposomes, generally resulting in equally high production levels of GM-CSF as with CD40-retargeted Ad-GM-CSF, never resulted in a maintained high expression of the DC activation markers (data not shown). Thus, not high GM-CSF levels *per se*, but rather the combination of CD40-mediated activation and GM-CSF production were responsible for the observed maintenance of CML-DC maturation subsequent to cytokine deprivation.

## DISCUSSION

Our finding of an enhanced and prolonged mature phenotype after transduction of CML-DC by CD40-targeted, Ad-mediated GM-CSF gene transfer may have implications for CML-DC-based immunotherapeutic approaches. Although previous studies showed the ability of CML-DC to prime CML-specific cytotoxic T lymphocytes (CTL) *in vitro* (Choudhury *et al*, 1997; Eibl *et al*, 1997), we were unable to detect any CTL responses in a recently conducted clinical pilot study in which three IFN-*α*-resistent CML patients in the late chronic phase of the disease were vaccinated with autologous, *in vitro* generated immature CML-DC (Ossenkoppele *et al*, 2003). In the same patients, we did observe postvaccination DTH responses against both CML and CML-DC and low-frequency Th responses to CML in peripheral blood. These results showed the feasibility of raising immune responses to CML through CML-DC-based vaccination, even in the presence of a large tumour burden, but at the same time indicated that the vaccine formulation needed to be optimised in order to activate tumor-specific CTL effectively. Numerous previous studies have pointed out the importance of proper DC activation in order to obtain long-lasting CTL responsiveness *in vivo*. Recent reports suggest that the use of immature DC for vaccination can induce peripheral T-cell tolerance, either through the induction of IL-10-producing regulatory T cells (Jonuleit *et al*, 2000), through the induction of T-cell anergy (Hawiger *et al*, 2001), or even through the elimination of specific CTL clones (Kurts *et al*, 1997). In light of these observations, it seems important to ensure a maintained mature phenotype of the adoptively transferred CML-DC in order to avoid eventual tolerance induction. A critical step in this context is the stimulation of immature DC by CD40L, inducing phenotypic and functional maturation of DC, and rendering DC capable of activating CTL (Schoenberger *et al*, 1998). The importance of this phenomenon for efficient tumour rejection has been confirmed by *in vivo* studies and shown to depend in large part on the production of IL-12 (van Mierlo *et al*, 2002). Our CD40-targeted approach of Ad transduction results both in the upregulation of costimulatory and adhesion molecules and in the release of IL-12, thus meeting the requirements for tumor-specific CTL activation. In addition, the transduction of CML-DC by CD40 targeted Ad vectors could serve as an ideal means to introduce cytokine transgenes for the induction of a more potent immune response and a prolonged survival of CML-DC. CD40 retargeting of Ad and simultaneous GM-CSF gene transfer resulted in a specific synergistic effect, characterised by high GM-CSF production levels and the significant elevation and prolonged expression of costimulatory and adhesion molecules on CML-DC. This observation indicates a possible clinical utility of CD40-retargeted Ad-GM-CSF transduction for the induction of a mature T-cell stimulatory phenotype in CML-DC, which may be expected to be stable over protracted periods of time after adoptive transfer.
